# A high-throughput, bead-based, antigen-specific assay to assess the ability of antibodies to induce complement activation^☆^

**DOI:** 10.1016/j.jim.2019.07.002

**Published:** 2019-10

**Authors:** Stephanie Fischinger, Jonathan K. Fallon, Ashlin R. Michell, Thomas Broge, Todd J. Suscovich, Hendrik Streeck, Galit Alter

**Affiliations:** a Ragon Institute of MGH, Harvard and MIT, Cambridge 02139, USA; b University of Duisburg-Essen, Essen 47057, Germany

**Keywords:** ADCD, Complement, Antibody-dependent effector function, High-throughput, Fc receptor

## Abstract

The complement system plays a critical role in innate immune defense against pathogens, both via non-specific direct pathogen recognition and killing or via antigen-specific indirect recruitment by complement fixing antibodies. While various assays for measuring complement activation have been developed, few provide a high-throughput, sample-sparing approach to interrogate the qualitative differences in the ability of antibodies to drive complement activation. Here we present a high-throughput, sample-sparing, bead-based assay to evaluate antigen-specific antibody-dependent complement activation against nearly any antigen. Optimization of buffer composition, kinetics of immune complex formation, as well as complement source all contribute critically to the development of a robust, highly flexible and high-throughput approach to analyze antibody-dependent complement deposition (ADCD). Thus, the optimized bead-based, antigen-specific assay represents a simple, highly adaptable platform to profile antibody-dependent complement activation across pathogens and diseases.

## Highlights

• Optimized flow-based assay for the detection of antibody-mediated complement deposition
• Robust, rapid and reproducible high-throughput bead-based assay applicable to various diseases, including HIV and influenza
• Lot controlled complement is a controlled source for exogenous complement that correlates with human complement activity

## 1 Introduction

Antibodies represent the primary correlate of protection following nearly all clinically approved vaccines and infections (Haynes et al., 2012; Sicca et al., 2018; Teo et al., 2016). Specifically, antibodies play a major role in host defense against pathogens by recognizing infected cells or the pathogen itself. While preventing pathogen entry is one potential mechanism by which antibodies may confer protection (i.e., neutralization), antibodies can also control and help clear infections through non-neutralizing immune effector functions (DiLillo et al., 2016; Lelièvre and Lévy, 2016). After antigen-binding, antibodies mediate these effector functions via interactions between their Fc domains and either Fc receptors (found on all innate immune cells) or components of the complement system. Fc/FcR interactions result in phagocytosis, induction of cell lysis, or degranulation, each of which has been associated with natural and vaccine-associated immunity (Excler et al., 2014; Markiewski and Lambris, 2007). However, beyond the direct recruitment of cellular innate immune functions, antibodies can also recruit complement to directly kill pathogens (Gunn and Alter, 2016) or deploy additional innate immune clearing activities via complement receptors (CRs) also found on most immune cells (Holers, 2014). With our emerging appreciation for the role of non-neutralizing antibodies in protection from infection, assays to selectively and specifically profile antibody-mediated immune activation have emerged. While several assays have been described for the analysis of antibody-mediated innate immune cellular activation (Ackerman et al., 2011), fewer high-throughput assays specifically and selectively probe the ability of antibodies to trigger the complement cascade.

The complement system is one of the first barriers of the innate immune system against pathogens, bridging innate and adaptive immunity (Markiewski and Lambris, 2007). The complement system consists of a tightly regulated network of soluble proteins in the blood, which can assemble to form a membrane-attack complex upon activation on the surface of cells or pathogens (Medof et al., 1982). Specifically, upon activation, complement proteins self-organize following a cascade of enzymatic reactions, resulting in the deposition of complement aggregates on target membranes. However, even in intermediate aggregates, complement proteins leverage host defense by facilitating opsinophagocytic clearance of pathogens and subsequent intracellular destruction (Beurskens et al., 2015; Sarma and Ward, 2011). However, emerging data also point to critical roles for the complement system beyond pathogen control, involved in tissue regeneration, development of the central nervous system, angiogenic network formation, and embryo implantation (Ricklin et al., 2010). Complement dysregulation on the other hand can result in tissue damage and ongoing inflammatory processes (Ballanti et al., 2013).

**Fig. 1:**
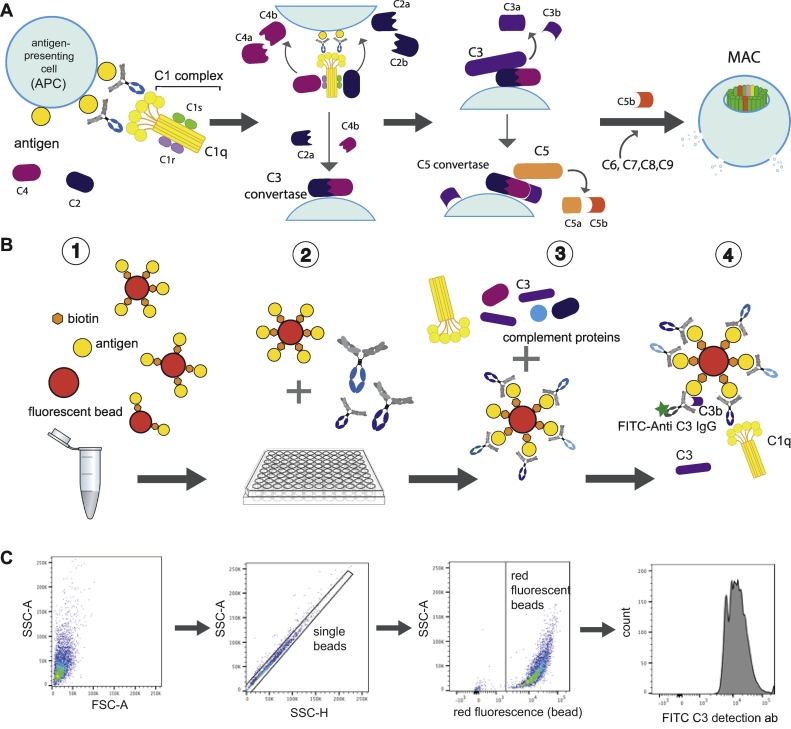
Antibody- dependent complement deposition assay procedure. A: The classical pathway is activated by C1q binding to immune complexes which causes a conformational change in the C1r:C1s-complex resulting in the activation of the enzymatic activity of C1r. C1r cleaves C1s which leads to cleavage of C4 and C2. C4b and C2b form the C3 convertase which facilitates cleavage of C3 into C3b which is deposited in the pathogen surface and together with C3 convertase forms the C5 convertase leading to cleave of C5. C5b triggers the formation of a pore by recruiting C6, C7, C8 and C9 to form the membrane attach complex (MAC) which leads to disruption of the cell membrane. B: The ADCD assay involves four major steps: First, the biotinylated antigen of interest is incubated with a fluorescent NeutrAvidin-coated bead, then the beads are washed and blocked with PBSA. The antigen-coupled beads are then added to the diluted antibody sample and incubated at 37 °C. After washing the beads, lyophilized guinea pig complement is reconstituted, diluted in veronal buffer, and incubated with the antibody-bead complex at 37 °C. After washing the beads, a FITC-conjugated anti-C3 detection antibody is added. C: Acquisition and flow gating strategy: the unfixed beads are acquired on a flow cytometer equipped with a high throughput sampler (HTS). Gates are drawn on single, red fluorescent particles, and complement deposition is reported as the MFI on the FITC channel.

Complement can be activated through three different pathways: the classical, the lectin and the alternative pathways. The classical pathway is activated via antibody-antigen complexes (Fig. 1A), mostly IgG and IgM (Nesargikar et al., 2012), the lectin pathway can be triggered via carbohydrates (Fujita, 2002) while the alternative pathway can be initiated spontaneously by membranes that fail to inhibit complement deposition (Harboe and Mollnes, 2008). Among the pathways, the classical pathway can be actively directed via vaccination, as it is activated by antibodies. Specifically, emerging data suggest that the degree of activation of the complement system is dependent on antibody isotype, subclass, affinity, and Fc-glycosylation, all of which evolve throughout an immune response (Holers, 2014; Lu et al., 2017; Prechl et al., 2010). For example, the immune response can select different antibody isotypes or subclasses, each with their own affinity for complement, with IgM having the greatest complement-fixing potential, followed by IgG3, IgG1, and then IgG2 and IgG4 (Coulie and van Snick, 1985). Upon IgG or IgM pathogen recognition, the C1q subunit of the C1 complex undergoes a conformational change, activating the C1r and C1s subunits and catalyzing the initiation of the cascade (Noris and Remuzzi, 2013). C1s cleaves the C4 and C2 proteins into two fragments each, two of which (C4b and C2a) then bind noncovalently to form the C3 convertase. This enzymatic complex in turn cleaves the complement C3 protein into the C3a and C3b fragments, representing the first step of amplification of the complement cascade. C3a release acts as an anaphylatoxin and a potent chemoattractant for immune cells such as neutrophils or monocytes. Conversely, the deposition of C3b represents the next step along the path to the recruitment of additional complement pathway components, ultimately aimed at assembling a membrane attack complex (MAC), and the formation of a pore resulting in cell lysis (Haas and van Strijp, 2007; Sarma and Ward, 2011). Importantly, along the path to the formation of the MAC complex, immune cells express a number of surface proteins that have the capacity to recognize and disassemble earlier steps along the antibody-induced complement cascade, preventing non-specific cellular destruction.

Emerging data point to a potentially critical role of complement in protection following various clinically approved vaccines (Frasch et al., 2009; Geurtsen et al., 2014) as well as experimental vaccines (McCoy et al., 2013), pointing to a role for vaccine-induced antibody-driven complement deposition as a potentially key predictor of immunity. While various assays have been developed to measure complement activity in human plasma, a sample-sparing, high-throughput assay could provide critical value for the evaluation and dissection of complement-activating antibodies. Assays using tumor cell lines expressing or adsorbed with antigens offer moderate throughput to analyze a limited number of antigens (Derer et al., 2014; Zhou et al., 2008). However, the use of specific target cells restricts the target antigen of interest and renders standardization more difficult. To overcome this gap, here we describe a high-throughput, sample-sparing, antibody-mediated complement activation assay that can be adapted to analyze immune responses to virtually any antigen. The antibody-dependent complement deposition assay described here is a bead-based assay using lyophilized guinea pig complement. This simple, reproducible and versatile assay is capable of screening large sample cohorts with different diseases across multiple antigens for complement-activating antibodies. Here we describe the optimization process and explain the nuances of the assay that can be easily adapted to study the immune response to various pathogens. This readily standardized assay provides a platform tool for the investigation of antibody-recruiting antibodies across pathogens and diseases.

## 2 Materials and methods

### 2.1 Samples and controls

Serum and plasma samples were collected from 2 healthy and 27 chronically HIV-infected individuals via the Ragon Institute of MGH, MIT and Harvard as a source of pathogen-specific antibodies. All subjects provided informed written consent. The study was conducted in accordance with the World Medical Association's Declaration of Helsinki and approved by the MGH Institutional Review Board. Additionally, polyclonal pooled HIV positive IgG (HIVIG, NIH AIDS Reagent Program) was used as a positive control at 5 mg/ml and pooled HIV negative IgG (IVIG, Sigma, I4506) was used at the same concentration as a negative control. Sample diluent without antibody was used to determine assay background. Samples were titrated to determine the optimal dilution, and a sample dilution of 1:10 was chosen for ADCD. This high level of dilution was expected given previously described studies that have highlighted the need for higher serum dilutions to detect complement activation compared to other antibody dependent assays (Ayoglu et al., 2014).

### 2.2 Preparation of antigen-coated beads

HIV gp120 (strain YU2) antigen (Immune Technology, IT-001-0027p YU-2) was biotinylated at lysine residues using sulfo-NHS-LC-biotin (Thermo Scientific, 21,935) according to the manufacturer's instructions. As an alternative antigen, influenza hemagglutinin (HA) (California H1N1 2009, Immunetech, IT-003-SW12ΔTMp) was used and biotinylated in the same way. To remove residual biotin after the reaction, antigens were buffer exchanged into phosphate buffered saline (PBS; Sigma-Aldrich, Saint Louis, USA) with Zeba Spin Desalting Columns (Thermo Scientific, 87766) with 40K MWCO according to the manufacturer's instructions. The biotinylated antigens were incubated, individually, with red 1.0 μm fluorescent neutravidin beads (Thermo Fisher, F8775) at 37 °C in a low-binding microcentrifuge tube (Corning, CLS3207). Beads were subsequently washed twice in 5% PBS- BSA, and then resuspended at a final dilution of 1:100 in 0.1% PBS-BSA. Antigen-coated beads were stored for up to 2 days at 4 °C and protected from light.

### 2.3 Complement detection

Different complement sources were tested. 1) Lyophilized guinea pig complement (Cedarlane, CL4051) was resuspended in 1 ml of distilled water, and was used as the first source of complement. 2) Human complement was collected in the form of plasma from ACD tubes from seronegative volunteers and used within 2 h of the blood draw. Specifically, blood was centrifuged at room temperature for 10 min at 1000 ×*g* and the supernatant was collected and re-centrifuged to remove platelets. 3) Lyophilized baby rabbit complement (Cedarlane, CL3441) was resuspended in 1 ml of distilled water. For heat-inactivation of complement, the complement was put on a heat block at 56 °C for different lengths of time. Afterwards, complement was centrifuged at 16,000 ×*g* for 5 min at 4 °C to remove any debris. Complement either from human serum or reconstituted guinea pig complement was then diluted 1:50, 200 μl of the final dilution was then added to assay wells. As dilution buffer, PBS, R10 (RPMI-1640, Sigma R0883 with 10% FBS, Sigma F2442), GVB (gelatin veronal buffer, Boston BioProducts, IBB-290X) or GVB++ (gelatin veronal buffer and additional Ca^2+^ and Mg^2+^, Boston BioProducts, IBB-300X) was used. Bead-based immune complexes were incubated with complement at 37 °C and then washed twice with 15 mM EDTA in PBS (Invitrogen, AM9260G). The deposition of complement was then assessed using anti-C3 antibodies. Specifically, fluorescein-conjugated goat anti-guinea pig complement C3 (MP Biomedicals, 0855385) was diluted 1:100 in PBS and 50 μl were added per well and incubated at room temperature for 15 min. For detection of human complement, a FITC-conjugated monoclonal detection antibody against human C3/C3b/iC3b (Cedarlane, CL7632F) was added at a 1:100 dilution in PBS. For comparison between different anti-human detection antibodies, polyclonal anti-C3 and monoclonal anti-C3 antibodies were used at a concentration of 0,5 μg/well (Quidel, A507 & A508). Baby rabbit complement was detected with a FITC-conjugated goat anti-rabbit polyclonal antibody against C3 (MP Biomedicals, 0855654) at a 1:100 dilution in PBS. Beads were then washed twice with 200 μl PBS by centrifugation at 2000*g* and resuspended in 100 μl PBS for acquisition. Optionally, stained bead-immune complexes were fixed in 100 μl 4% PFA (Santa Cruz, sc-281692) for 20 min, then spun down at 2000*g* and resuspended in 100 μl PBS. A total of 50 μl of the fixed beads were then analyzed by flow cytometry on the BD LSR II with a high throughput sampler (HTS) for the detection of anti-C3 complement antibody. Events were gated on single beads and bead positive events, meaning a positive signal in the bead color channel. As the final readout, the median fluorescence intensity of all bead positive events in the FITC channel were reported. Results were analyzed using FlowJo 10 and visualized using GraphPad Prism7.

### 2.4 Visualization of complement-opsonized antibody-coated beads

For the visualization of successful bead coupling and detection, the Amnis ImageStreamX imaging flow cytometer was used combining the phenotyping abilities of flow cytometry with the detailed imaging of microscopy. This system captures an image of each bead as it passes through the stream, allowing for quantification of beads and fluorescence as well as visualization of the actual bead. Pictures were taken in the bright field, FITC, and PerCP-Cy5.5 channels of the instrument. Amnis-collected images were analyzed using the IDEAS software package in order to determine overlap of the bead and secondary antibody fluorescent colors.

### 2.5 Analysis

Statistical analysis was performed using GraphPad Prism 7. A non-parametric Spearman's correlation was used, values were considered statistically significant if two-tailed p-value < 0.05.

## 3 Results

### 3.1 Assay overview

The high throughput, antibody-dependent complement deposition (ADCD) assay can be split into four steps. The steps include: 1) the attachment of antigen to fluorescent beads, 2) the formation of immune complexes, 3) addition of complement, and 4) detection of complement C3 deposition via an anti-C3 antibody (Fig. 1B). The beads are then acquired and analyzed for C3 deposition via flow cytometry (Fig. 1C).

### 3.2 Detecting complement deposition

**Fig. 2:**
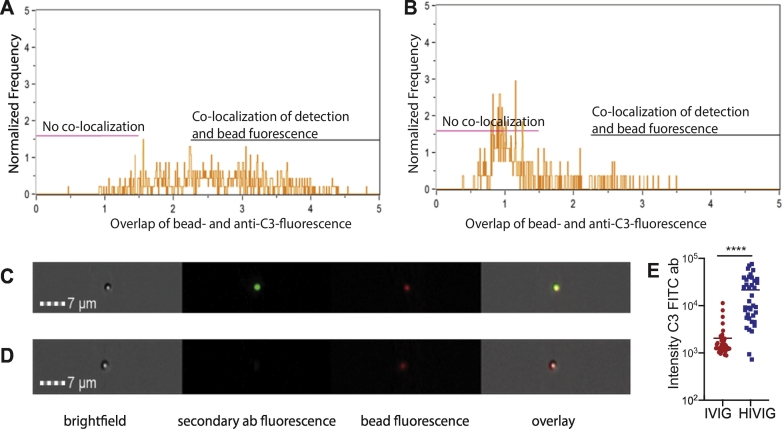
Image stream analysis confirms the detection of complement deposition on fluorescent beads. 5 mg/ml of HIVIG (pooled IgG from HIV-infected individuals as positive control) and IVIG (pooled IgG from healthy individuals as negative control) were used as samples for the detection of complement-activating antibodies against the HIV gp120 antigen. After completing the bead-based complement deposition assay, samples were run on the Amnis ImageStreamX imaging flow cytometer in order to quantify the fluorescence of the fluorescent bead and the detection antibody. The overlay of bead fluorescence and secondary antibody color is depicted in panels A and B. Panels C and D show representative microscopy pictures. A: HIVIG shows a broad overlap of the two fluorescent channels with the spread of co-localization on the x-axis representing the differences in FITC fluorescence. B: IVIG sample shows a low amount of co-localization between bead and secondary antibody fluorescence. C: HIVIG as a sample gives both bead and complement fluorescence, that co-localize as shown in the overlay. D: IVIG does not induce complement deposition on the HIV-bead and therefore only the bead fluorescence is visible. E: Intensity of secondary antibody fluorescence for IVIG and HIVIG for 40 beads is shown. Mann-Whitney test was performed, ****p < 0.0001.

To initially determine whether complement deposition could be selectively and specifically observed on antigen-coupled beads in the presence of sero-positive pools of antibodies, an Amnis ImageStreamX imaging flow cytometer was used to visualize the binding of the detection antibody to C3 complement following incubation with pools of HIV-positive pools of polyclonal IgG (HIVIG) or HIV-negative pools of polyclonal IgG (IVIG). Following gating on red fluorescence, the level of C3 deposition was visualized (Fig. 2A and B). The x-axis represents the differences in C3-FITC fluorescence detected by the secondary antibody, with higher positivity in the presence of the HIVIG compared to the IVIG (Fig. 2A and B). These data highlight the specific nature of C3 deposition in the presence of HIV-specific antibodies. To further visualize the overlap of the C3 binding to the bead, an Imagestream analysis was performed. Specifically, the overlap of C3 deposition was visualized across beads. A clear overlap of the FITC anti-C3 secondary fluorescence and the red bead fluorescence was observed in the overlapping image (Fig. 2C and D). The difference between detection of complement via FITC in HIVIG and IVIG samples was highly significant (Fig. 2E). Thus, the C3-bead based deposition assay is specific and allows the simple identification of antigen-specific antibodies able to drive complement deposition.

### 3.3 Antigen-bead coupling

**Fig. 3:**
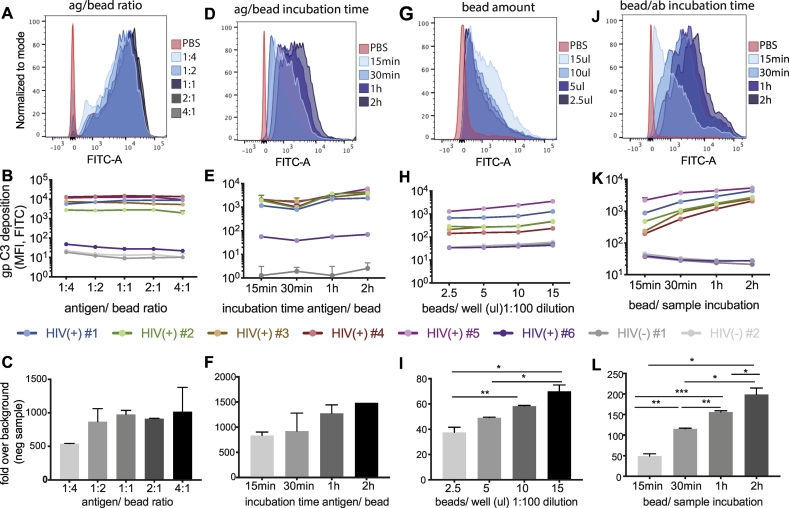
Optimization of the bead coupling step and formation of immune complexes. The step of coupling of beads and antigen was optimized by testing 4–5 different conditions each for the ratio of antigen to beads, incubation time of antigen and beads, the amount of beads added per well, and the antibody sample incubation time. Six HIV-positive samples and two HIV-negative samples were tested at a 1:10 dilution for all of the optimization experiments. The histograms show results for the HIV positive sample #2 under the different conditions and the PBS control. Bar graphs indicate mean ± SD of technical duplicates. A–C: Different antigen to bead ratios from 1:4 to 4:1 were tested. D-F: Beads and biotinylated antigen were co-incubated for different time spans: 15 min, 30 min, 1 h and 2 h at 37 °C. G–I: The amount of beads per well was optimized by adding different volumes of the 1:100 bead dilution after coupling and washing to each well. Either 2.5, 5, 10 or 15 μl of a 1:100 bead dilution were added per well. J–L: The incubation time of antigen-coupled beads with antibody sample to form immune complexes was varied from 15 min up to 2 h at 37 °C.

As an initial step, antigen coupling to the bead was optimized. The influence of antigen to bead ratios on C3 deposition was examined. A wide range of ratios were explored, ranging from four times more bead volume than antigen (μg antigen:μl beads = 1:4) to four times more antigen than beads (μg antigen:μl beads = 4:1). There was no significant difference in C3 deposition between the different ratios in the raw flow cytometric plots (Fig. 3A) as well as in the analyzed data (Fig. 3B and C). Although these ratios may vary by antigen, here we elected to use a 1:1 ratio of bead to antigen for further optimization of the assay.

To next define whether antigen coupling time also influenced ADCD activity, the level of C3 deposition was next probed, using a 1:1 bead-to-antigen ratio, following antigen-bead coupling times ranging from 15 min to 2 h at 37 °C. Variation was observable in C3 deposition with beads conjugated for longer periods of time (Fig. 3D and E), with a 2 h incubation showing the greatest level of C3 deposition. Importantly, no statistically significant signal to noise differences were observed across the incubation times tested (Fig. 3F). Thus, the 2 h antigen incubation time was selected moving forward.

### 3.4 Optimizing bead input

To next define the impact of bead number on C3 deposition, varying amounts of beads were added per well, always maintaining a 1:100 bead dilution. While the addition of 2.5–10 μl of beads (4.6 × 10^7^–1.8 × 10^8^ beads) gave similar levels of ADCD, the addition of 15 μl (2.7 × 10^8^) of beads gave the most robust shift in ADCD activity (Fig. 3G). Negative samples remained strongly negative at all bead input numbers (Fig. 3H). Conversely, a clear dose effect was observed across the HIV-positive donor samples (Fig. 3I). Differences in ADCD across samples could be visualized across bead input numbers above 2.5 μl (4.6 × 10^7^), highlighting the utility of using bead numbers of 9 × 10^7^ (5 μl) or higher. Thus, for consistency, an intermediate volume of 10 μl/well (1.8 × 10^8^ beads/well) was used in subsequent experiments.

### 3.5 Optimizing serum sample incubation time

In order to determine the optimal incubation time for diluted serum samples and antigen-coated beads, samples were incubated with beads for 15 min to 2 h at 37 °C. Strikingly, complement activity could be visualized as early as 15 mins post immune complex incubation (Fig. 3J and K). Moreover, while qualitative differences among samples were most clearly observable at lower incubation times, maximal complement activity was clearly observed after a 2 h incubation (Fig. 3L). Interestingly, background ADCD activity decreased with time, highlighting the increasing specificity of the assay with longer incubation times. Importantly, the hierarchy of ADCD activity across the test samples did not change significantly over the different incubation time course, suggesting that although greater resolution between sample activity could be discerned at 30 min, a 2 h incubation period provides both increased signal to noise and an opportunity to tease out qualitative differences in antibody-mediated complement depositing activity. Thus, for all follow-up experiments, a 2 h sample incubation time was used.

### 3.6 Guinea pig complement serves as a stable source of complement

**Fig. 4:**
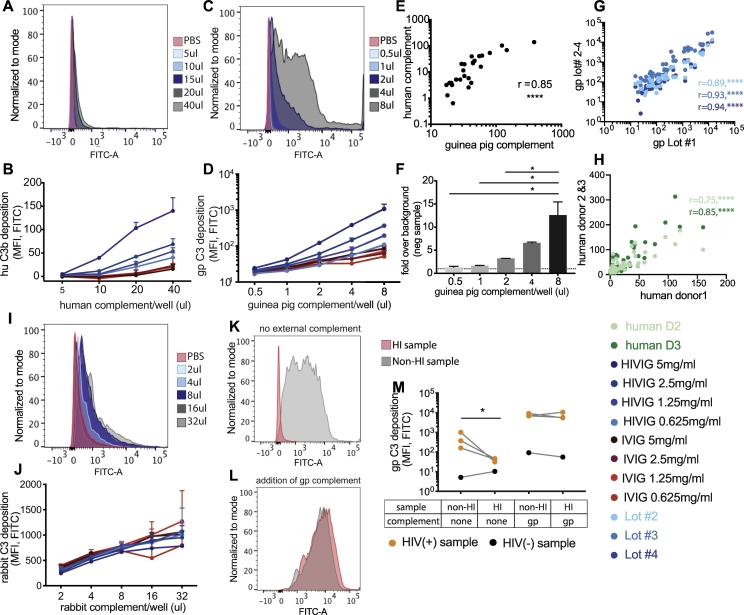
Comparison of complement from different species and optimization of the complement incubation step. The ADCD assay was run using human (A, B, and H), guinea pig (C–G, K–M), or rabbit (I–J) complement, together with HIV gp120 as antigen and plasma or purified IgG from HIV-positive and HIV-negative subjects. A different, species-specific detection antibody was used for each source of complement. A, C, I: The histograms indicate results for the 5 mg/ml HIVIG sample using the indicated amounts of complement per well or a PBS control. B, D, J: Line graphs show C3 deposition results over a range of complement amounts, for the indicated HIVIG and IVIG concentrations. Point and error bars indicate mean ± SD of technical triplicates. E: C3 deposition results using 20 μl/well human complement or 4 μl/well guinea pig complement were correlated. Dots represent data for each HIVIG and IVIG concentration tested in panels B and D. F: Bar graph shows the calculated signal (C3 deposition using 5 mg/ml HIVIG) to noise (C3 deposition using 5 mg/ml IVIG) ratios for the indicated amounts of added guinea pig complement. Bars represent the mean ± SD of technical triplicates. The dotted line indicates a signal-to-noise ratio of 1. One way ANOVA was performed on all groups, *p < 0.0332. G: Correlation plot shows the pairwise correlation between four guinea pig complement lot numbers across HIV positive and negative samples. H: Correlation plot depicts comparison of three human plasma donors against four concentrations of HIVIG and IVIG over different amounts of added human plasma as complement source (same set up as in 4B). All correlations were performed as two-tailed Spearman correlations. ****, p < 0.0001 K and L: Histograms depict complement deposition results for an HIV-positive plasma sample with or without prior heatinactivation of the sample, either in the absence (K) or presence (L) of 4 μl/well exogenous guinea pig complement. M: C3 deposition results are shown for HIV-positive and HIV-negative plasma samples tested with or without prior heat-inactivation of the samples, and either with or without 4 μl/well exogenous guinea pig complement. Statistics were performed using a paired *t*-test. *p > 0.0332. Differences between HI or non-HI sample were not statistically significant by paired t-test.

While the complement system is highly conserved across species (Nonaka and Kimura, 2006), previous studies have explored various sources of complement including human (Borrow et al., 2001), rabbit (Borrow et al., 2001), and guinea pig (Kim et al., 2017). To gain greater insights into the comparability of distinct sources of complement in this ADCD assay, we specifically explored differences in complement deposition using human (species matched) or lyophilized, stable, batch-controlled guinea pig complement. Human complement was sourced from fresh human plasma, and FITC-conjugated detection antibodies were used to match the species of each complement source. Low but detectable ADCD activity was observed using human complement (Fig. 4A and B). Conversely, high levels of complement deposition were observed in the presence of guinea pig complement (Fig. 4C and D). Despite the differences in the magnitude of C3 deposition across complement sources, complement deposition was highly correlated across assays using the fresh human and reconstituted guinea pig complement (Fig. 4E), highlighting the utility of lot-controlled guinea pig complement as a robust source of complement. Further, titration of guinea pig complement input revealed that the addition of 4 μl of guinea pig complement per well was sufficient to observe separation across positive and negative antibody-sources (Fig. 4D). Increased signal was observed at higher input complement concentrations, with associated increases in signal to noise ratios (Fig. 4F). Thus, adding 4 μl of guinea pig complement per well was considered sufficient to induce a strong signal that was five-fold over background. Moreover, different lots of guinea pig complement were tested using this optimal amount, and the results were found to be highly correlated and comparable (Fig. 4G). Along these lines, three different human donors were tested and the complement deposition results showed a high correlation (Fig. 4H). Background complement deposition was measured using IVIG or HIV-negative plasma, but we also showed that human C3-deficient and guinea pig C4-deficient plasma as complement source results in no signal (Supplemental Fig. 2). Rabbit complement was tested as a potential source of complement as well (Fig. 4I), but this was not considered a reliable source because no differences between positive and negative signal were detectable (Fig. 4J).

### 3.7 Heat inactivation of serum samples results in a marginal improvement in assay performance

Given the presence of complement in plasma and serum samples, we next aimed to determine whether inactivation of sample-complement was essential for assay performance. Because the complement system is known to be heat sensitive (Güven et al., 2014; Montefiori, 2017), plasma samples were heat-inactivated at 56 °C for 30 min. The ability of antibodies to fix complement was then assessed in the presence or absence of exogenous complement. In the absence of additional complement, heat inactivation decreased complement deposition (Fig. 4K), suggesting that residual serum complement can drive complement fixation to different extents depending on the sample (Fig. 4M). However, in the setting of exogenous complement activation, the signal was magnified (Fig. 4L), masking any effect of residual heat inactivation (Fig. 4M). Therefore, although residual complement in plasma samples only showed a marginal effect on ADCD signal in the presence of external complement, heat inactivation of test serum samples is recommended for this assay. This additional step can reduce the effects of residual complement in plasma samples, which might vary across donors because of sample storage conditions, freeze-thaw cycles, and processing protocols (Gao et al., 2018).

### 3.8 Selecting optimal media and buffers

**Fig. 5:**
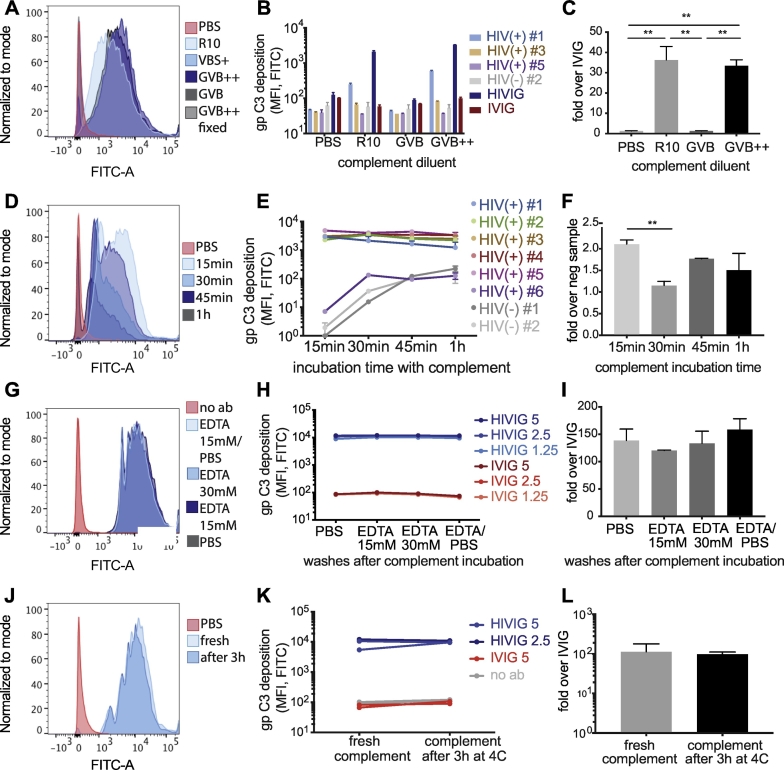
Optimization of the complement incubation step. The ADCD assay was run using HIV gp120-coupled beads, 4 μl/well guinea pig complement, and plasma or purified IgG from HIV-positive and HIV-negative subjects. A–C: The reconstituted complement was diluted in different-test buffers. The histogram (A) shows C3 deposition results for HIV-positive sample #1 or a PBS control together with complement diluted in the indicated buffers. The bar graph (B) shows C3 deposition results using each complement diluent and three HIV-positive plasma samples, one HIV-negative plasma sample, HIVIG, and IVIG. The bar graph in (C) depicts the signal (C3 deposition using 5 mg/ml HIVIG) to noise (C3 deposition using 5 mg/ml IVIG) ratio for the different-test buffers. D–F: Guinea pig complement (4 μl/well, diluted in GVB++) was added to the immune-complexed beads and incubated for different time spans ranging from 15 min to 1 h at 37 °C. The histogram (D) shows results for HIV positive sample #2 for each complement incubation time or PBS control. Graph (E) shows results for six HIV-positive and two HIV-negative plasma samples across complement incubation times. The bar graph (F) depicts the signal (C3 deposition for HIV-positive plasma sample #5) to noise (C3 deposition for HIV-negative plasma sample #2 for each complement incubation time. G–I: After complement incubation, the impact of different EDTA concentrations in the wash buffer was investigated against PBS washes. The histogram (G) shows the C3 deposition results with a PBS control sample or 5 mg/ml HIVIG for each washing condition. Graph (H) shows the C3 deposition results for 1.25–5 mg/ml HIVIG or IVIG sample for each test wash buffer. The bar graph (I) depicts the signal (C3 deposition for 5 mg/ml HIVIG) to noise (C3 deposition for 5 mg/ml IVIG) ratios for each assay wash buffer. J–L: The short-term stability of reconstituted guinea pig complement was tested in the ADCD assay. The histogram (J) shows C3 deposition results for PBS control or 5 mg/ml HIVIG sample tested with freshly reconstituted guinea pig complement or complement that had been reconstituted 3 h earlier and stored at 4 °C. Graph (K) shows extended results from the same experimental setup using HIVIG and IVIG samples. The bar graph (L) depicts the signal (C3 deposition for 5 mg/ml HIVIG) to noise (C3 deposition for 5 mg/ml IVIG) ratios for each assay condition. Differences in results between using freshly-reconstituted or 3 h-old complement were not statistically significant by t-test (L). For all analyses, a one-way ANOVA was used to compared differences across >2 groups and a paired *t*-test was used to test differences across 2 groups. *p < 0.05, **p < 0.005, ***p < 0.0005.

Next, to further probe the stability of the guinea pig complement-based ADCD assay, we initially tested whether different media could improve the signal to noise ratio of the assay. Specifically, a number of distinct media were compared including phosphate-buffered saline (PBS), RPMI media with 10%. Fetal bovine serum (R10), Gelatin veronal buffer (GVB, containing 0.1% gelatin) and GVB++ (GVB with additionally added calcium and magnesium). PBS and GVB were expected to yield the lowest signal due to the lack of Ca^2+^ and Mg^2+^, whereas GVB++ and R10 have additional Ca^2+^ and Mg^2+^. As expected, the ADCD assay performed best in the presence of R10 and GVB++ (Fig. 5A and B). While diluting complement in GVB++ showed the greatest separation between positive and negative controls, R10 also showed a similar trend (Fig. 5C).

To next define the optimal time span for complement incubation, a time course was evaluated ranging from 15 min to 1 h. The most dramatic signal to noise separation was observed after 15 min (Fig. 5D and E). While the positive serum samples showed equivalent levels of complement fixation over the entire time course, non-specific complement deposition increased over time (Fig. 5E). Therefore, a 15-minute complement incubation time was selected for the assay to maximize the signal to noise ratio and enhance assay performance (Fig. 5F).

Previous data suggested that EDTA may block complement activation, and particularly affect C1q deposition (Schwaiger et al., 2014). To therefore determine whether a wash buffer containing EDTA could improve assay performance by stopping complement activity, wash buffers containing different amounts of EDTA (15 mM and 30 mM of EDTA in PBS) were compared against PBS (Fig. 5G). However, the addition of exogenous EDTA did not increase background or alter the ADCD levels significantly (Fig. 5H and I), highlighting the negligible effects of wash buffer composition.

### 3.9 Guinea pig complement can be used for a period of time after reconstitution

The supplier's instructions state that lyophilized guinea pig complement must be used immediately upon reconstitution (Cedarlane Labs, 2018). To assess how quickly reconstituted complement loses its activity, complement was tested immediately or 3 h after reconstitution. No difference was observed between complement used immediately or 3 h following reconstitution with storage at 4 °C (Fig. 5J-L), suggesting that complement should not lose activity during assay setup.

### 3.10 No difference in plasma and serum in the ADCD assay

**Fig. 6:**
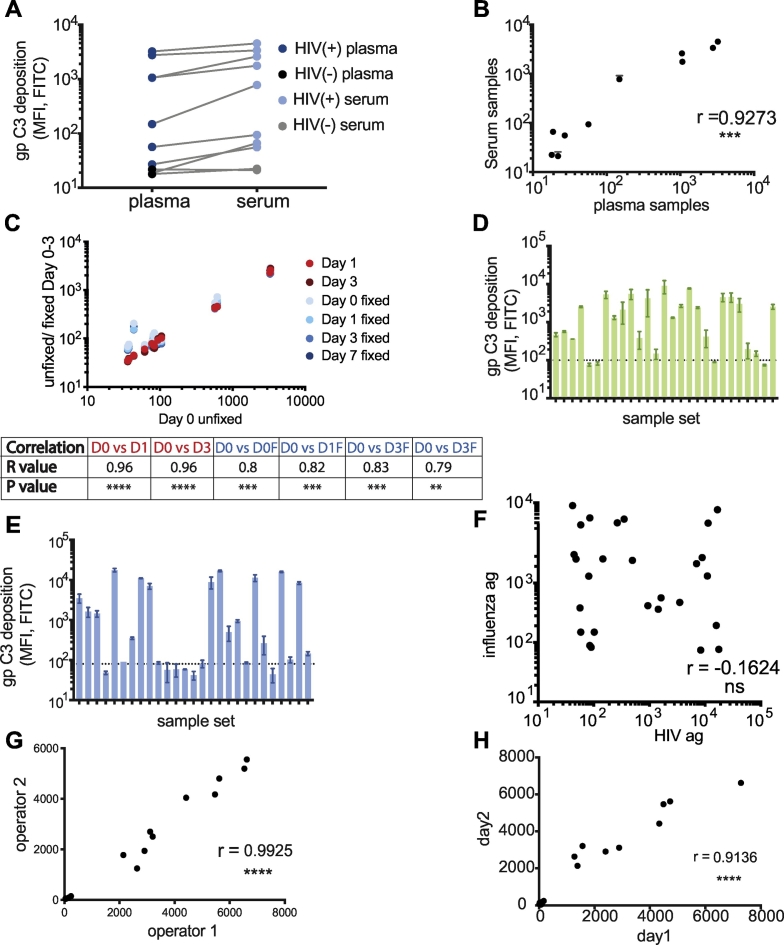
Assessment of the versatility and reproducibility of the ADCD assay. A-C: The ADCD assay was tested for versatility by comparing plasma and serum samples prepared from the same whole blood draws from 8 HIV-positive and 2 HIV-negative individuals. These samples were tested using the standard assay protocol, HIV gp120-coupled beads, a 1:10 sample dilution, and guinea pig complement. Shown are (A) the individual C3 deposition results for each test sample and (B) the correlation of C3 deposition results for plasma versus serum samples from each subject. Additional assay plates were set up in parallel using the same plasma samples, the beads were either resuspended in PBS or fixed with 4% PFA at the end of the assay, and then the beads were acquired on a flow cytometer on the day of the assay (Day 0) or up to 7 days later. (C) The correlation of C3 deposition results for each assay condition and time point are shown versus unfixed beads acquired on Day 0. D–F: A sample set of 27 HIV-positive samples was tested against an (D) influenza HA antigen (H1N1, California, 2009) and (E) HIV gp120 antigen (YU2). Bars indicate mean ± SD of technical replicates, and dotted line represents assay background (PBS control). The correlation of C3 deposition results for the sample set for HIV gp120 and influenza HA antigens is shown in (F). G: Two independent operators tested 10 HIV-positive and 2 HIV-negative plasma samples in the ADCD assay using HIV gp120-coupled beads. The correlation of C3 deposition results obtained between operators is shown in (G). H: A single operator tested 9 HIV-positive and 2 HIV-negative plasma samples in the ADCD assay using HIV gp120-coupled beads twice on separate days. The correlation of C3 deposition results obtained between the two assay runs is shown in (H). For all analyses, a one-way ANOVA was used to compared differences across >2 groups, a paired t-test was used to test differences across 2 groups, and correlations were assessed using a two-tailed Spearman correlation: ***p < 0.0005, ****p < 0.0001.

Some antibody functional assays perform more robustly using serum compared to plasma (Schwenk et al., 2010). Thus, we next aimed to determine whether ADCD activity differed across serum and plasma samples prepared from the same blood draws. While marginally increased levels of ADCD were observed using serum samples, these differences were not statistically significant (Fig. 6A). Moreover, C3 deposition results for serum and plasma samples correlated highly, suggesting that both serum and plasma can be used in the ADCD assay (Fig. 6B).

### 3.11 Optimizing time to acquire data by flow cytometry

To determine whether the ADCD assay must be analyzed immediately by flow cytometry, we evaluated the quality of results in fixed or unfixed samples analyzed immediately or several days post-assay setup. Importantly, no variation was observed after 1 or 3 days in unfixed samples, suggesting that the complement-bound beads are highly stable during that time (Fig. 6C). Additionally, only minimal changes were observed in the presence of fixation, but the signal was highly reproducible after bead acquisition 1, 3, or even 7 days later (Fig. 6C). Thus, the ADCD complexes are highly stable in the presence or absence of fixatives and can be analyzed on a flow cytometer up to 1 week after setting up the assay.

### 3.12 The ADCD assay is highly versatile and reproducible

**Supplemental Fig. S1:**
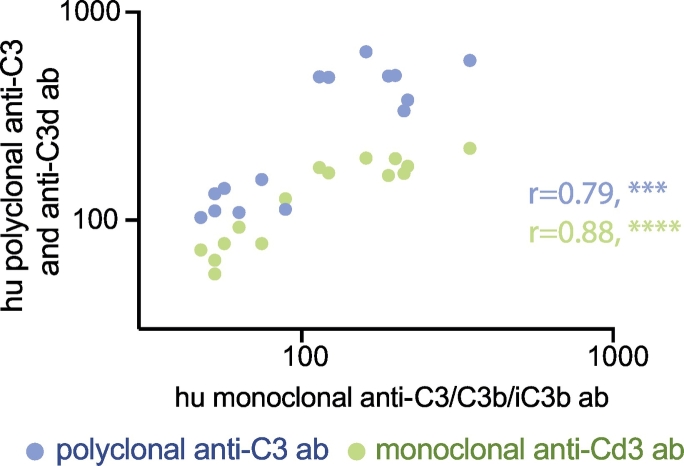
Comparison of different complement anti-C3 detection antibodies. The correlation plot depicts the complement deposition assay results using FITC-conjugated monoclonal anti-human C3/C3b/iC3b, monoclonal anti-human C3d, and polyclonal anti-C3 antibodies. Human plasma (20 μl/well) was used as a complement source together with HIV gp120-coupled beads and 0.5–5.0 mg/ml HIVIG or IVIG sample. Correlations were performed using a two-tailed Spearman correlation; ***, p < 0.0005, ****, p < 0.0001.

Because the ADCD assay is conducted on antigen-conjugated beads, nearly any antigen may be theoretically coupled to beads, offering a highly flexible platform for the analysis of complement fixing antibodies. Thus, to test the flexibility of the platform, the utility of an additional, non-HIV-derived antigen was tested in the assay. Influenza hemagglutinin (HA, California H1N1 2009) was used to detect responses against influenza in our HIV-positive sample set. Because the plasma samples were drawn between 2010 and 2015, reactivity to HA antigen from a 2009 influenza strain was assessed. As expected, all samples exhibited some level of HA-specific ADCD activity, although the activity was variable across the sample group (Fig. 6D). Importantly, the HA-specific pattern was distinct from that observed for HIV YU-2 gp120-specific antibody responses (Fig. 6E and F). Thus, as expected, the ADCD platform can be used to explore antigen-specific antibody-dependent complement-fixing activities across a wide range of diseases, that may be regulated in vastly different ways during infection or following vaccination. We also evaluated different FITC-conjugated monoclonal and polyclonal detection antibodies against C3, C3b and C3d, and found the results for each to be highly correlated (Supplemental Fig. S1). This indicates that the polyclonal detection antibody against full-length C3 that was used to optimize this assay can rank samples in terms of complement-activating antibodies as well as a C3b- or C3d-specific monoclonal antibody.

### 3.13 Testing the robustness of the assay

Finally, to investigate the robustness of this assay, reproducibility was investigated across multiple users and across assay runs using the same set of plasma samples. Strikingly concordant ADCD levels were observed across operators that ran the samples on the same day (r = 0.99, p < 0.001) (Fig. 6G). Similarly, ADCD experiments run on the same samples over a two-day period also showed robust concordance (Fig. 6H). Thus, the assay is not only highly versatile, enabling the interrogation of antigen-specific complement fixing functions across a wide range of antigens, the assay is also robust, offering a simple and reproducible platform for the analysis of a critical antibody effector function.

## 4 Discussion

The complement system is part of the innate immune system, providing a first line of defense against pathogens, but also contributing to the regulation of adaptive immunity (Carroll, 2004). Several assays have been developed to specifically profile the ability of antibodies to drive complement-induced killing of pathogens. However, the development of a high-throughput, sample-sparing assay, able to dissect the role of antibodies that drive complement activation to any antigen of interest, could support the analysis of the impact of complement broadly across vaccines, diseases, and health. Here we present a bead-based complement deposition assay that requires minimal sample input and can be run at high-throughput, with short incubation times and lot-controlled lyophilized complement, even days after the assay is run.

The critical role of complement in anti-pathogen activity is most clearly illustrated in complement-deficient populations (de Córdoba et al., 2011). Primary C3 deficiencies, while rare, are associated with increased susceptibility to bacterial infections that primarily manifest in early childhood, marked by pneumonia and meningitis (Fijen et al., 1994). Moreover, individuals with C3 deficiencies are more prone to develop glomerulonephritis (Kosaka et al., 2013) and lupus (Pickering et al., 2000), pyogenic and respiratory infections (Skattum et al., 2011). For example, subjects with complement deficiencies have a significantly greater risk for meningococcal infections, specifically related to the inability of antibodies to fix complement and drive bacteriolysis (Platonov et al., 2003).

Beyond complement deficiencies, complement has been linked to protection across a broad array of infectious diseases, including against bacterial infections such as pertussis (Geurtsen et al., 2014) and meningococcal disease (Lewis and Ram, 2014), but has also been linked to protection against malaria (Boyle et al., 2015; Khan et al., 2015). In viral infections, complement plays an important role in antibody-mediated neutralization of dengue (Shresta, 2012), protection against West Nile virus (Vogt et al., 2011; Wu et al., 2015) and influenza infection (O'Brien et al., 2011). In contrast, the role of complement in HIV pathogenesis has been more controversial. Complement has been implicated in both promoting HIV infection (Stoiber et al., 2001) as well as in driving enhanced viral lysis in early stages in HIV infection (Liu et al., 2014). More specifically, HIV can acquire complement regulatory proteins (CRPs) from the host membrane during budding, and the presence of CRPs rescues HIV from complement-mediated virolysis leading to complement opsonization of the viral surface (Bánki et al., 2005). This opsonized virus has been shown to enhance susceptibility of monocytes, macrophages and dendritic cells to HIV infections (Pruenster et al., 2005). Whether complement opsonization on the HIV surface leads to clearance of the virus or promotes infection remains elusive. However, across all of these infections, disparate assays have been used to interrogate the role of antibody mediated complement activation, rendering it difficult to compare or identify conserved complement functions across pathogens.

In the context of vaccination, the serum bactericidal antibody assay (SBA) was selectively designed to probe the role of complement-fixing antibodies in vaccine-induced immunity. The SBA defined complement as a correlate of protection following meningococcal C conjugate vaccination (Goldschneider et al., 1969). Similarly, the in vitro opsonophagocytic assay (OPA) measuring bacterial growth restriction (Romero-Steiner et al., 1997), the MAC detection by ELISA (Jeon et al., 2014), the iC3b detection by lateral flow (Schramm et al., 2015) and other bead-based assays (Ayoglu et al., 2014) have all been used to profile vaccine-induced antibody-mediated complement activity. However, many of the existing assays are time consuming and require bacterial plating and growth (Romero-Steiner et al., 1997), although newer techniques have reduced some of the bacterial work (Guttormsen et al., 2009). In context of the RV144 HIV-1 vaccine trial, Perez et al. performed a Luminex bead-based assay to measure complement activation using human plasma as a complement source (Perez et al., 2017). While this multiplexed assay allows for the measurement of C3d complement deposition over several antigens, it requires special readout equipment and cost-intensive Luminex beads. Many of the aforementioned assays are still hampered by the use of bacteria as the primary antigen-presenting source, thereby limiting the application of these assays since they cannot reliably be used to dissect and quantitate antibody-mediated responses involved in viral/fungal infections or autoimmune/oncological conditions. Here whole pathogens, components, or epitope-scaffolds can be individually coupled to beads, with simply coupling adaptions, to fully interrogate the response. Furthermore, the requirement for fresh human plasma as the gold standard in SBA reduces throughput and may introduce variation (Bash et al., 2014), even though we have shown that human complement is comparable across donors. Conversely, the high-throughput assay described here can be adapted for profiling of almost any antigen, including glycan-, lipid-, or protein-based antigens, that may be coupled to a bead in a sample-sparing, robust, and reproducible manner.

Antibody-mediated complement fixation may lead to both full pathogen destruction or phagocytic clearance of opsonized targets (Beurskens et al., 2015). In our assay, it is unclear if the profiled antibodies lead to the ultimate formation of a MAC complex or simply direct immune clearance. Given the high-throughput nature and the flexibility of the assay, additional analyses may be performed to further dissect the impact of complement-activating antibodies in the context of the disease of interest. For example, the addition of neutrophils, monocytes, or dendritic cells (DCs) following complement deposition on immune complexes, would allow the dissection of the specific innate immune cells or even complement receptors, that may drive indirect immune clearance. Additionally, the contribution of individual complement receptors, or the collaboration with Fc-receptors may be examined using blocking reagents or knock-out innate immune cells.

Our data showed that activation of guinea pig and human complement by human antibodies are highly correlated, and guinea pig complement can therefore be used to measure antibody-dependent complement deposition in human plasma. Previous studies have shown that complement component C1q is highly conserved across evolution and that the activity as well as Fc-binding ability of human and guinea pig complement are comparable (Dodds and Petry, 1993; Sasaki and Yonemasu, 1984). The evolution of complement components C1q and C3 can be traced back to the split between mammalian and nonmammalian genes, suggesting high levels of similarity between species (Nonaka and Kimura, 2006). The sequence homology of the C1q binding protein between guinea pig (Cavia porcellus) and *Homo sapiens* via BLAST showed 83% identity. It was shown that human IgM can activate the guinea pig complement system, but only with complexed antigen rather than soluble antigen (van der Zee et al., 1986). Conversely, other data suggest that the ability of complement to bind hexameric or pentameric antibodies varies between mouse and guinea pig (Collins et al., 2002), however both species respond. Moreover, the bactericidal activity of human antibodies against Meningococcus varies across species, as rabbit complement showed higher bactericidal activity (Zollinger and Mandrell, 1983). These data indicate that while C1q is conserved across species, some variation between the ability of human and guinea pig complement system to be activated is described. Despite these differences, the utility of lot-controlled guinea pig complement offers unique control over this critical reagent. Comparison of guinea pig and human complment highlighted clear detection, correlation, and reproducibility across samples. Thus, lyophilized guinea pig complement offers a highly controlled source of complement for the strict comparison of antibody-complement fixing changes across sample sets. Conversely, the use of human complement could provide interesting measures of changes in both antibodies and complement activity across populations, offering valuable insights into complement responsiveness in various disease states or transplant rejection (Stites et al., 2015). However, with simple adaptions in detection, non-human primates, rabbits, ferrets, etc., could also be used, offering broader opportunities to examine antibody/complement changes across diseases.

The beads used in this assay offer a static surface upon which antigens may be added to explore complement deposition, but also antigen-density and even bead size (cell size, bacteria size, or virus size) may influence the nature of complement activation. While a medium sized (1 μm diameter) bead was used in the assay described here, commercially available beads of distinct sizes may be used to mimic target complement size. Additionally, while the assay was optimized for maximal antigen saturation, variable levels of antigen may be coupled to the surface of the beads. In the HIV field for example, the importance of antigen structure (monomer versus native HIV trimer envelope structure) that may be targeted by distinct antibodies (Richard et al., 2018) has been emerging. In our assay, uniform antigen structures that are difficult to control on the surface of an infected or transfected cell, may be tethered to the surface of a bead. Thus, the ADCD assay described here offers a controlled antigen arraying technology for the specific analysis of humoral immune responses of interest.

The optimized antibody-dependent complement assay represents a simple, sample-sparing, high-throughput assay for the analysis of either polyclonal or monoclonal complement activation that can be used across various diseases and human sample types. Given the robustness of the assay, this bead- and flow-based assay offers a unique standardized approach for the assessment of antigen-specific antibody-dependent complement activation in human clinical samples across pathogens and diseases.

The following are the supplementary data related to this article.

**Supplemental Fig. S2:**
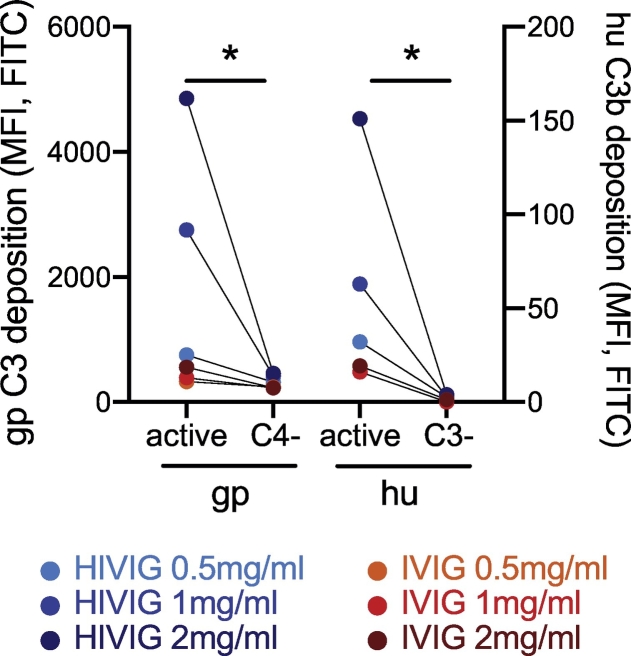
C3/C4-deficient plasma does not elicit complement deposition. Active and C4-deficient guinea pig complement (4 μl/well) as well as active and C3-deficient human complement (20 μl/well) were tested in the ADCD assay with HIV gp120-coupled beads and 0.5–2.0 mg/ml HIVIG or IVIG sample. A FITC-conjugated, species-specific detection antibody was used for each complement source. The paired Wilcoxon test was performed, *p < 0.0332.

## Funding

This work was supported by NIH, United States (AI080289, AI102660-01, AI129797-01); Bill and Melinda Gates Foundation, United States (OPP1114729, OPP1146996, OPP1032817); Massachusetts General Hospital ECOR, United States; Harvard University Center for AIDS Research, United States (P30 AI060354-02); Ragon Institute of MGH, MIT, and Harvard, United States

## Declaration of Competing Interests

None.
